# Incidence risk of peripheral edema in cancer patients treated with PD-1/PD-L1 inhibitors: A PRISMA guideline systematic review and meta-analysis

**DOI:** 10.1097/MD.0000000000030151

**Published:** 2022-09-09

**Authors:** Yuan Tian, Alan Huang, Mei Tian, Kaiyong Wang, Qi Dang, Caiqing Zhang, Hongmei Liu, Junyan Zhao, Xiaowei Yang, Chi Zhang, Liang Guo, Feng Chen

**Affiliations:** a Radiotherapy Department of Oncology, Shandong Second Provincial General Hospital, Jinan, Shandong 250023, P. R. China; b Department of Oncology, Jinan Central Hospital, the Hospital Affiliated with Shandong First Medical University, Jinan, Shandong 250013, P. R. China; c Respiratory Department, Affiliated Hospital of Shandong University of Traditional Chinese Medicine, Jinan, Shandong 250014, P. R. China; d Department of Respiratory and Critical Care Medicine, The People’s Hospital of Yuncheng County, Heze, Shandong 274799, P. R. China; e Phase I Clinical Trial Center, Shandong Cancer Hospital and Institute, Shandong First Medical University and Shandong Academy of Medical Sciences, Jinan, Shandong 250012, P. R. China; f Department of Respiratory and Critical Care Medicine, Shandong Second Provincial General Hospital, Shandong University, Jinan, Shandong 250023, P. R. China; g Radiotherapy Department, The First Affiliated Hospital of Shandong First Medical University & Shandong Provincial Qianfoshan Hospital, Jinan, Shandong 250014, P. R. China; h Nursing Department, The First Affiliated Hospital of Shandong First Medical University & Shandong Provincial Qianfoshan Hospital, Jinan, Shandong 250014, P. R. China; i Department of Hepatobiliary Intervention, Beijing Tsinghua Changgung Hospital, School of Clinical Medicine, Tsinghua University, Beijing, China; j Cardiology Department, The Second Hospital, Cheeloo College of Medicine, Shandong University, Jinan, Shandong 250033, P. R. China; k Radiotherapy Department of Oncology, The Fourth People’s Hospital of Jinan City, The Third Affiliated Hospital of Shandong First Medical University, Jinan, China; l Department of Thoracic surgery, Shandong Cancer Hospital and Institute, Shandong First Medical University and Shandong Academy of Medical Sciences, Jinan 250117, China.

**Keywords:** cancer, clinical trial, meta-analysis, PD-1/PD-L1, peripheral edema

## Abstract

To elucidate the relationship between peripheral edema and programmed cell death-1/programmed cell death ligand 1 (PD-1/PD-L1) inhibitors, the meta-analysis was performed.

**Method:**

Following the guidelines of Preferred Reporting Items for Systematic Reviews and Meta-analyses, all-grade and grade 3-5 of peripheral edema data extracted from clinical trials were taken into account for the final comprehensive assessments.

**Results:**

Twenty-seven PD-1/PD-L1-related clinical trials with peripheral edema data were collected. Compared with chemotherapy (PD-1/PD-L1 vs chemotherapy), the risk of developing peripheral edema for all-grade was much lower (odds ratio [OR] = 0.36, 95% confidence interval [CI]: [0.23, 0.56], *Z* = 4.55 [*P* < .00001]). When PD-1/PD-L1 plus chemotherapy were compared with chemotherapy, no significant analysis results for all-grade was found (OR = 1.15, 95% CI:[0.93, 1.44], *I*^2^ = 25%, *Z* = 1.27 [*P* = .20]). Similar risk trends could also be found when the incidence risk of peripheral edema for grade 3–5 was evaluated. No obvious publication bias was identified throughout the total analysis process.

**Conclusion:**

The effect of PD-1/PD-L1 inhibitor on the risk of developing peripheral edema was weaker than that of chemotherapy, and the combination with chemotherapy slightly increased the incidence risk of developing peripheral edema without statistical significance.

## 1. Introduction

Cancer immunotherapies, including immune checkpoint inhibitors (ICIs) and adoptive cell therapy,^[1]^ have played a significant role on the treatment of cancer patients.^[1–3]^ In clinical work, programmed cell death-1/programmed cell death ligand 1 (PD-1/PD-L1) inhibitors are the most common type of ICIs used in the treatment of solid malignancies.^[3,4]^ PD-1/PD-L1 inhibitors can block the interaction between T cells and tumor cells by binding to the immune checkpoint proteins expressing on T cells and tumor cells.^[1–4]^ Due to their unique mechanism of eliminating tumor tells, PD-1/PD-L1 inhibitors can induce a series of specific immune-related adverse events (irAEs) which almost affect any organs or systems.^[5–7]^

The majority of adverse events had attracted more and more attention from multidisciplinary clinicians and researchers, and relevant guidelines had been drafted to manage these irAEs.^[7,8]^ However, little information about PD-1/PD-L1 inhibitors related peripheral edema could be found online and such information was reported mostly in the form of case reports.^[9,10]^ For cancer patients, there are many factors that can lead to peripheral edema, such as liver and kidney disease, chemotherapy, malnutrition, targeted therapy, and immunotherapy drugs, which further increases the difficulty of defining the cause of peripheral edema and giving clinical symptomatic treatment.

With the increasing variety of antitumor treatment regimens combined with PD-1/PD-L1 inhibitors, more and more peripheral edemas have gradually been reported.^[11–42]^ But systematic reviews and meta-analysis about PD-1/PD-L1 inhibitors induced peripheral edema had rarely been found. In order to evaluate the incidence risk of PD-1/PD-L1 inhibitors related peripheral edema, this meta-analysis was carried out.

## 2. Methods

This systematic review and meta-analysis was carried out following the guidelines of the Preferred Reporting Items for Systematic Reviews and Meta-analyses (PRISMA).^[43]^

### 2.1. Types of enrolled studies

First, to meet the initial inclusion criteria, the study were limited to randomized, open-label, controlled clinical trials including the data of PD-1/PD-L1 inhibitors for cancer patients. Second, Phase III clinical trials of solid tumors investigating PD-1/PD-L1 inhibitors were preferentially selected. Other phase clinical trials would be carefully screened for selection and placed in an alternative options. Third, the eligible clinical trials must include one of the following data: all-grade or grade 3–5 peripheral edema. Finally, clinical trials of hematological malignancies were excluded decisively. In order to collect as many articles as possible, the control groups were not strictly restricted to a certain therapeutic drug or intervention.

### 2.2. Search strategy

We followed the guidelines of the PICOS (participants, interventions, comparisons, outcomes) as recommended by the Cochrane Collaboration.^[43]^ A PubMed search was conducted using the search terms: “neoplasm,” “cancer,” “precancer,” “pre- cancer,” “malignant,” “premalignant,” “tumor,” “tumour,” “PD-1,” “PD-L1,” “PD1,” “PDL1,” “nivolumab,” “Opdivo,” “pembrolizumab,” “Keytruda,” “Imfinzi,” “MK-3475,” “atezolizumab,” “Tecentriq,” “MPDL3280A,” “avelumab,” “Bavencio,” “durvalumab,” “camrelizumab,” and “BMS-963558,”

Studies were only enrolled when they were published in English between July 9, 2013 and December 25, 2021. Three authors were responsible for the qualification screening of all retrieved papers and data gathering. In the case of repeated reports of the same clinical trial, only the convincing one was selected for the final analysis. The baseline characteristics of all enrolled clinical trials were summarized in (Table 1).

**Table 1:** Baseline characteristics of included clinical trials (N = 27).

No.	Reference			NCT number	Phase	Drug name	Treatment regimen	First-line treatment	Tumor type	Involving patients	Any treatment related peripheral edema	Treatment related grade 3–5 peripheral edema
PD-1/PD-L1 vs chemotherapy												
1	Barlesi et al^[11]^			NCT02395172 (JAVELIN Lung 200)	III	Avelumab (PD-L1)	Avelumab vs Docetaxel	No	NSCLC	758	33	3
2	Hida et al^[12]^			NCT02008227 (OAK)	III	Atezolizumab (PD-L1)	Atezolizumab vs Docetaxel	No	NSCLC	101	17	0
	Rittmeyer et al^[13]^									1187	136	4
3	Herbst et al^[14]^			NCT01905657 (KEYNOTE-010)	II/III	Pembrolizumab (PD-1)	Pembrolizumab 2 mg/kg vs Docetaxel	No	NSCLC	648	26	0
	Herbst et al^[14]^						Pembrolizumab 10 mg/kg vs Docetaxel	No	NSCLC	652	25	0
4	Borghaei et al^[15]^			NCT01673867 (CheckMate057)	III	Nivolumab (PD-1)	Nivolumab vs Docetaxel	No	NSCLC	555	36	1
5	Brahmer et al ^[16]^			NCT01642004 (CheckMate017)	III	Nivolumab (PD-1)	Nivolumab vs Docetaxel	No	NSCLC	260	10	0
6	Powles et al^[17]^			NCT02853305 (KEYNOTE-361)	III	Pembrolizumab (PD-1)	Pembrolizumab vs Gemcitabine + Csplatin or Carboplatin	Yes	UC	644	72	3
7	Bellmunt et al^[18]^			NCT02256436 (KEYNOTE-045)	III	Pembrolizumab (PD-1)	Pembrolizumab vs (Paclitaxel, Docetaxel, or Vinflunine)	No	UC	521	66	2
8	Cohen et al^[19]^			NCT02252042 (KEYNOTE-040)	III	Pembrolizumab (PD-1)	Pembrolizumab vs (Methotrexate, Docetaxel or Cetuximab)	No	HNSCC	480	4	1
PD-1/PD-L1 + chemotherapy vs chemotherapy												
1			West et al^[20]^	NCT02367781 (IMpower130)	III	Atezolizumab (PD-L1)	Atezolizumab + Carboplatin + Nab-paclitaxel vs Carboplatin + Nab-paclitaxel	Yes	NSCLC	705	90	5
2			Emens t al^[21]^	NCT02425891 (IMpassion130)	III	Atezolizumab (PD-L1)	Atezolizumab + Nab-paclitaxel vs Placebo + Nab-paclitaxel	Yes	TNBC	890	141	7
			Schmid et al^[22]^								85	6
3			Mittendorf et al^[23]^	NCT03197935 (IMpassion031)	III	Atezolizumab (PD-L1)	Atezolizumab + Chemotherapy vs Placebo + Chemotherapy	No	TNBC	331	45	1
4			Langer et al^[24]^	NCT02039674 (KEYNOTE-021)	II	Pembrolizumab (PD-1)	Pembrolizumab + Carboplatin + Pemetrexed vs Carboplatin + Pemetrexed	Yes	NSCLC	121	9	0
5			Zhou et al^[25]^	NCT03134872 (CameL)	III	Camrelizumab (PD-1)	Camrelizumab + Carboplatin + Pemetrexed vs Carboplatin + Pemetrexed	No	NSCLC	412	32	0
6			Abreu et al^[26]^	NCT02578680 (KEYNOTE-189)	III	Pembrolizumab (PD-1)	Pembrolizumab + Pemetrexed + platinum-based drug vs Placebo + Pemetrexed + Platinum-based drug	Yes	NSCLC	607	135	2
			Horinouchi et al^[27]^							40	7	0
			Gadgeel et al^[28]^							607	117	2
			Gandhi et al^[29]^							607	104	1
7			Powles et al^[17]^	NCT02853305 (KEYNOTE-361)	III	Pembrolizumab (PD-1)	Pembrolizumab + Gemcitabine + Csplatin or Carboplatin vs Gemcitabine + Csplatin or Carboplatin	Yes	UC	691	92	2
8			Yang et al^[30]^	NCT03707509 (CAPTAIN-1st)	III	Camrelizumab (PD-1)	Camrelizumab + Gemcitabine + Cisplatin vs Placebo + Gemcitabine + Cisplatin	Yes	NPC	263	12	N/A
9			Rudin et al^[31]^	NCT03066778 (KEYNOTE-604)	III	Pembrolizumab (PD-1)	Pembrolizumab + Etoposide + Platinum vs Placebo + Etoposide + Platinum	Yes	SCLC	446	44	0
Others												
1		Nathan et al^[32]^		NCT03070392	III	Pembrolizumab (PD-1)	Tebentafusp vs (Pembrolizumab, Ipilimumab, or Dacarbazine)	Yes	Melanoma	356	69	N/A
2		Gutzmer et al^[33]^		NCT02908672 (IMspire150)	III	Atezolizumab (PD-L1)	Atezolizumab + Vemurafenib + Cobimetinib vs Placebo + Vemurafenib + Cobimetinib	Yes	Melanoma	511	43	0
3		Ribas et al^[34]^		NCT02027961	I	Durvalumab (PD-L1)	Durvalumab 3 or 10 mg/kg + Dabrafenib + Trametinib vs Durvalumab 10 mg/kg + Trametinib (concurrent)	No	Melanoma	46	10	0
		Ribas et al^[34]^					Durvalumab 3 or 10 mg/kg + Dabrafenib + Trametinibvs. Durvalumab 10 mg/kg + Trametinib (sequential)			48	15	0
		Ribas et al^[34]^					Durvalumab 10 mg/kg + Trametinib (concurrent) vs Durvalumab 10 mg/kg + Trametinib (sequential)			42	13	0
4		Gogas et al^[35]^		NCT03273153 (IMspire170)	III	Atezolizumab (PD-L1)	Atezolizumab + Cobimetinib vs Pembrolizumab	Yes	Melanoma	436	58	2
5		Sullivan et al^[36]^		NCT01656642	I	Atezolizumab (PD-L1)	Atezolizumab + Vemurafenib vs Atezolizumab + Cobimetinib +Vemurafenib	Yes	Melanoma	56	23	0
6		Choueiri et al^[37]^		NCT03141177CheckMate 9ER	III	Nivolumab (PD-1)	Nivolumab + Cabozantinib vs Sunitinib	Yes	RCC	640	62	1
7		Motzer et al^[38]^		NCT01668784 (CheckMate025)	III	Nivolumab (PD-1)	Nivolumab vs Everolimus	No	RCC	803	73	2
8		Motzer et al^[39]^		NCT02684006 (JAVELIN Renal 101)	III	Avelumab (PD-L1)	Avelumab + Axitinib vs Sunitinib	Yes	RCC	873	84	3
9		Powles et al^[17]^		NCT02853305 (KEYNOTE-361)	III	Pembrolizumab (PD-1)	Pembrolizumab vs Pembrolizumab + Gemcitabine + Csplatin or Carboplatin	Yes	UC	651	76	3
10		Bellmunt et al^[40]^		NCT02450331 (IMvigor010)	III	Atezolizumab (PD-L1)	Atezolizumab vs Observation	No	UC	787	57	1
11		Lee et al^[41]^		NCT02952586 (JAVELIN Head and Neck 100 trial)	III	Avelumab (PD-L1)	Avelumab + Chemoradiotherapy vs Placebo + Chemoradiotherapy	Yes	HNSCC	692	30	16
12		Kang et al^[42]^		NCT02267343 (ATTRACTION-2)	III	Nivolumab (PD-1)	Nivolumab vs Placebo	No	GC/GEJC	491	38	1
13		Herbst et al^[14]^		NCT01905657 (KEYNOTE-010)	II/III	Pembrolizumab (PD-1)	Pembrolizumab 2 mg/kg vs Pembrolizumab 10 mg/kg	No	NSCLC	682	9	0

GC/GEJC = gastric or gastroesophageal junction cancer, HNSCC = head and neck squamous cell carcinoma, NPC = nasopharyngeal carcinoma, NSCLC = nonsmall cell lung cancer, PD-1 = programmed cell death-1, PD-L1 = programmed cell death ligand 1, RCC = renal cell carcinoma, SCLC = small cell lung cancer, TNBC = triple-negative breast cancer, UC = urothelial cancer.

### 2.3. Evaluation of study quality and publication bias

Publication bias was assessed by using the Egger regression test, while the quality of all included trials was screened with the help of the Newcastle-Ottawa scale, which was proposed by the Cochrane Collaboration.^[43–47]^ The quality assessments of all enrolled clinical trials were also finished by the above 3 reviewers. The quality assessment contents were listed as follows: random sequence generation, allocation concealment, blinding of participants and personnel, blinding of outcome assessment, incomplete outcome data, and selective outcome reporting. All these contents were evaluated together, and the evaluation results would be summarized in a single graph. The Harbord test was used for checking publication bias for all enrolled clinical trials.^[48]^ *P* value < .05 was considered to be indicative publication bias.

### 2.4. Exposure and outcome of interest

Basic characteristics of enrolled studies, including the first author’s name, year of publication, trial number, trial title, the phase of clinical trial, the specific name of the anti-PD-1/PD-L1 agent, treatment regimens, the status of previous therapies, tumor type, the number of participants, the number of all-grade peripheral edema events (rate), and the number of grade 3–5 peripheral edema events, were collected and summarized in (Table 1).

### 2.5. Assessment of heterogeneity and statistical analysis

Heterogeneity of all the eligible studies was evaluated by Cochrane Q statistic and the I^2^ statistic,which was advocated by Higgins and colleagues.^[43,47]^ The degree of heterogeneity grading was evaluated by the range of *I*^2^ values.^[43,47]^ Heterogeneity was regarded as low, moderate, or high according to *I*^2^ values < 25%, 25% to 50%, and >50%, respectively. Odds ratio (OR), and the corresponding 95% confidence interval (CI) would be calculated by random effect.^[49]^ *P* value of <.05 was considered as the cutoff value for statistical significance. In order to clarify the relationship between peripheral edema and PD-1/PD-L1 inhibitors, we performed a large number of subgroup analyses based on the type of tumor, the treatment regimen, and the specific administered drug. The software (Review Manager 5.3) was adopted for data consolidation and analysis. Statistical tests were all 2-sided.

## 3. Results

### 3.1. Literature search results

**Figure 1.**
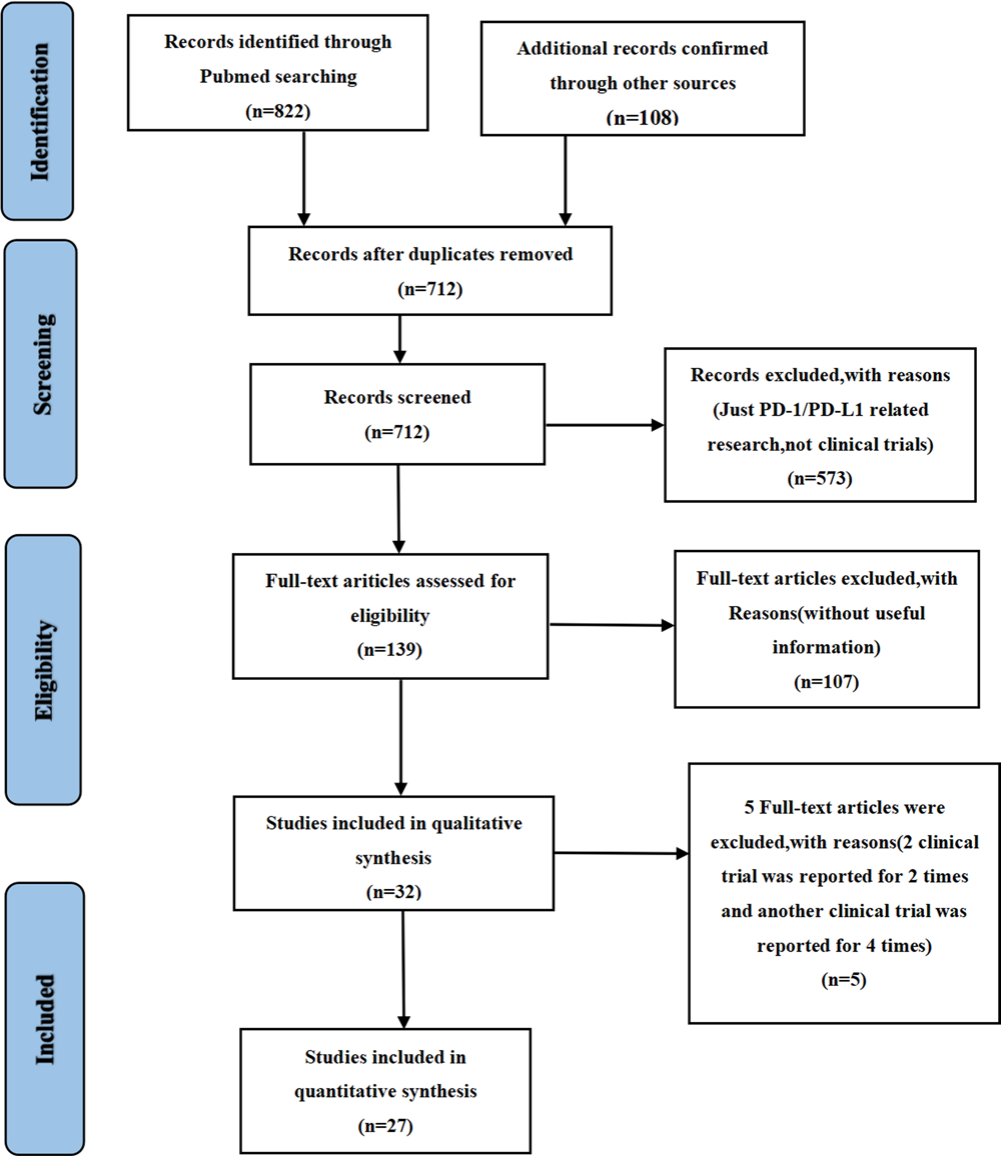
A PRISMA flow diagram of the screening process of the study.

**Figure 2.**
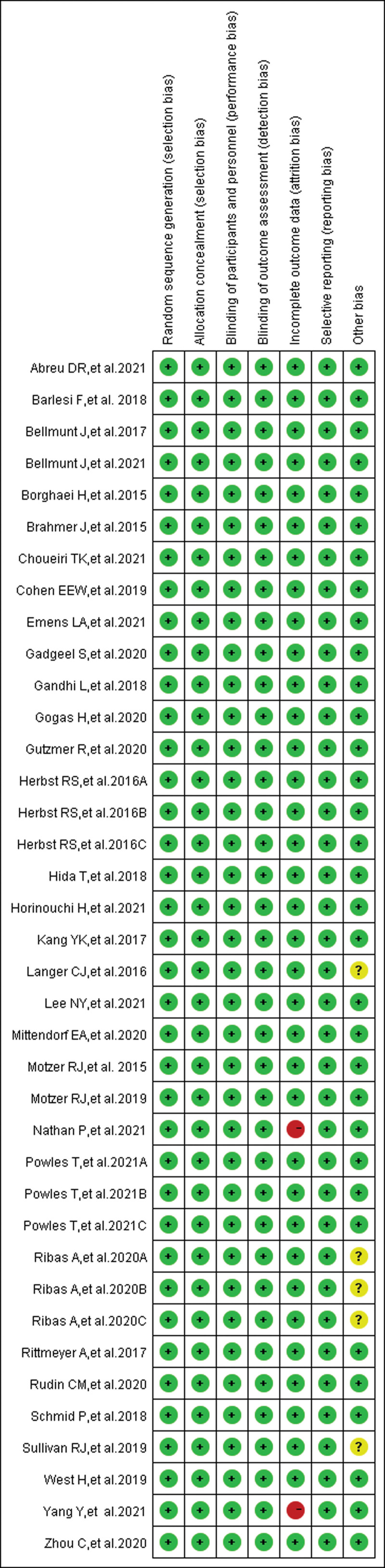
Risk of bias summary: review authors’ judgments about each risk of bias item for each included study.

According to our initial electronic search on the PubMed website, a total of 822 studies investigating PD-1/PD-L1 inhibitors for cancer patients were identified. 32 studies met our preliminary selection criteria,^[11–42]^ of which 27 articles were selected for the meta-analysis.^[11,13–21,23–26,30–42]^ The results of 3 clinical trials had been repeatedly reported for several times: OAK (n = 2),^[12,13]^ IMpassion130 (n = 2),^[21,22]^ and KEYNOTE-189 (n = 4).^[26–29]^ When such repeated reports for the same clinical trials were noted, only the convinced one was selected for the meta-analysis. The PRISMA flow diagram of the screening process for the clinical trials was shown in (Fig. 1), while the quality assessments of all included studies were provided in (Fig. 2).

### 3.2. Characteristics of identified trials

The basic characteristics of the 32 eligible studies were screened and summarized in (Table 1).^[11–42]^ Most of those studies (n = 28) reported in the form of phase III clinical trials,^[11–13,15–23,25–33,35,37–42]^ while only 2 trials were phase I.^[34,36]^ The rest 2 clinical trials were a phase II/III clinical trial and a phase II clinical trial.^[14,24]^ 15 clinical trials (reported in 18 literature) were PD-1 inhibitors involved,^[14–19,24–32,37,38,42]^ while the rest 12 clinical trials (reported in 14 literature) were about PD-L1 inhibitors.^[11–13,20–23,33–36,39–41]^ As shown in (Table 1), most of the studies were related to pembrolizumab (8 clinical trials),^[14,17–19,24,26–29,31,32]^ atezolizumab (7 clinical trials),^[12,13,20–23,35,36,40]^ nivolumab (5 clinical trials),^[15,16,37,38,42]^ and avelumab (4 clinical trials).^[11,33,39,41]^ Few studies were involved in camrelizumab (2 clinical trials),^[25,30]^ and durvalumab (1 clinical trial).^[34]^

Nine tumor types were found among the enrolled clinical trials, of which 10 trials were nonsmall cell lung cancer (NSCLC).^[11–16,20,24–29]^ The other tumors types were listed as follows: melanoma (involving 5 clinical trials),^[32–36]^ triple-negative breast cancer (involving 3 clinical trials),^[21–23]^ renal cell carcinoma (involving 3 clinical trials),^[37–39]^ urothelial cancer (involving 3 clinical trials),^[17,18,40]^ head and neck squamous cell carcinoma (HNSCC) (involving 2 clinical trials),^[19,41]^ nasopharyngeal carcinoma(NPC) (involving 1 clinical trial),^[30]^ small cell lung cancer(SCLC) (involving 1 clinical trial)^[31]^ and gastric or esophageal junction cancer (GC/GEJC) (involving 1 clinical trial).^[42]^ Among the 27 clinical trials, PD-1/PD-L1 inhibitors were prescribed as the first-line treatment option in 14 clinical trials,^[17,20–22,24,26–33,35–37]^ while patients with previous anticancer treatment before PD-1/PD-L1 inhibitors were found in the other 13 clinical trials.^[11–16,18,19,23,25,34,38,40,42]^ All the clinical trials were divided into 2 groups according to the treatment regimen as shown in (Table 1): Group A (PD-1/PD-L1 vs Chemotherapy),^[11–19]^ Group B (PD-1/PD-L1 + Chemotherapy vs Chemotherapy).^[20–31]^

### 3.3. Risk of bias

The risk bias of all the enrolled studies was shown in Figure 2,^[11–42]^ while the publication bias, evaluated by the Harbord test, was displayed in the form of funnel plots in the supplemental digital contents (Supplemental Digital Contents 1 to 4, http://links.lww.com/MD/H61).^[11,13–21,23–26,30,31]^

### 3.4. The Incidence risk of peripheral edema for all-grade

**Figure 3.**
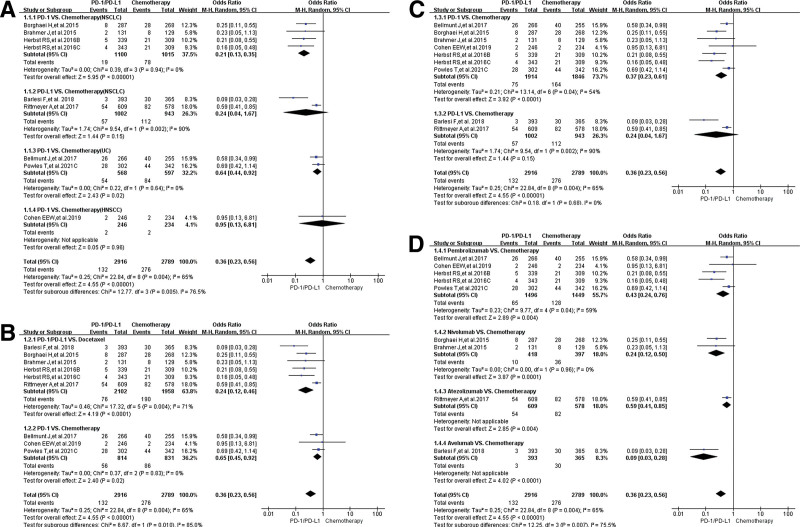
Forest plots of all-grade peripheral edema for Group A (PD-1/PD-L1 vs chemotherapy). (A) The risk of peripheral edema for all-grade evaluated by random effect model: subgroup analysis was carried out based on tumor types. (B) The risk of peripheral edema for all-grade evaluated by random effect model: subgroup analysis was carried out based on chemotherapy regimen (Docetaxel or chemotherapy). (C) The risk of peripheral edema for all-grade evaluated by random effect model: subgroup analysis was carried out based on immunosuppressants types (PD-1 or PD-L1). (D) The risk of peripheral edema for all-grade evaluated by random effect model: subgroup analysis was carried out based on specific immunosuppressive drugs. PD-1 = programmed cell death-1, PD-L1 = programmed cell death ligand 1.

Among all the enrolled clinical trials, peripheral edema of all-grade was reported in 27 clinical trials.^[11–42]^ Only 16 clinical trials were used in the final meta-analysis.^[11,13–21,23–26,30,31]^ Compared with chemotherapy (group A), the risk of developing peripheral edema for all-grade in PD-1/PD-L1 involved subgroup was significantly lower (OR = 0.36, 95% CI: [0.23, 0.56], *I^2^* = 65%, *Z* = 4.55 [*P <* .00001]; Fig. 3).^[11,13–19]^ Subgroup analysis indicated a statistically significant difference in the risk of developing peripheral edema among different tumor types, especially for HNSCC (Chi^2^ = 12.77, df = 3 [*P* = .005]; Fig. 3A). Similar risk trend could also be seen when the control group was divided by chemotherapy regimen (Fig. 3B). However, when PD-1 subgroup was compared with the PD-L1 subgroup, no statistical difference was found (OR: 0.36 vs 0.24; Chi^2^ = 0.18, df = 3 [*P* = .68]; Fig. 3C). Atezolizumab appeared to have a higher risk of developing peripheral edema when subgroup analyses were performed by specific drug name (OR = 0.59, 95% CI:[0.41, 0.85], Fig. 3D). Subgroup analysis suggested that the high heterogeneity (*i*^2^ = 65%, Fig. 3A) might be mainly attributable to the 2 lung cancer-related clinical trials (JAVELIN Lung 200 and OAK).^[11–13]^ No obvious publication bias was found in the corresponding funnel plots (Supplemental Digital Content 1, http://links.lww.com/MD/H61).

**Figure 4.**
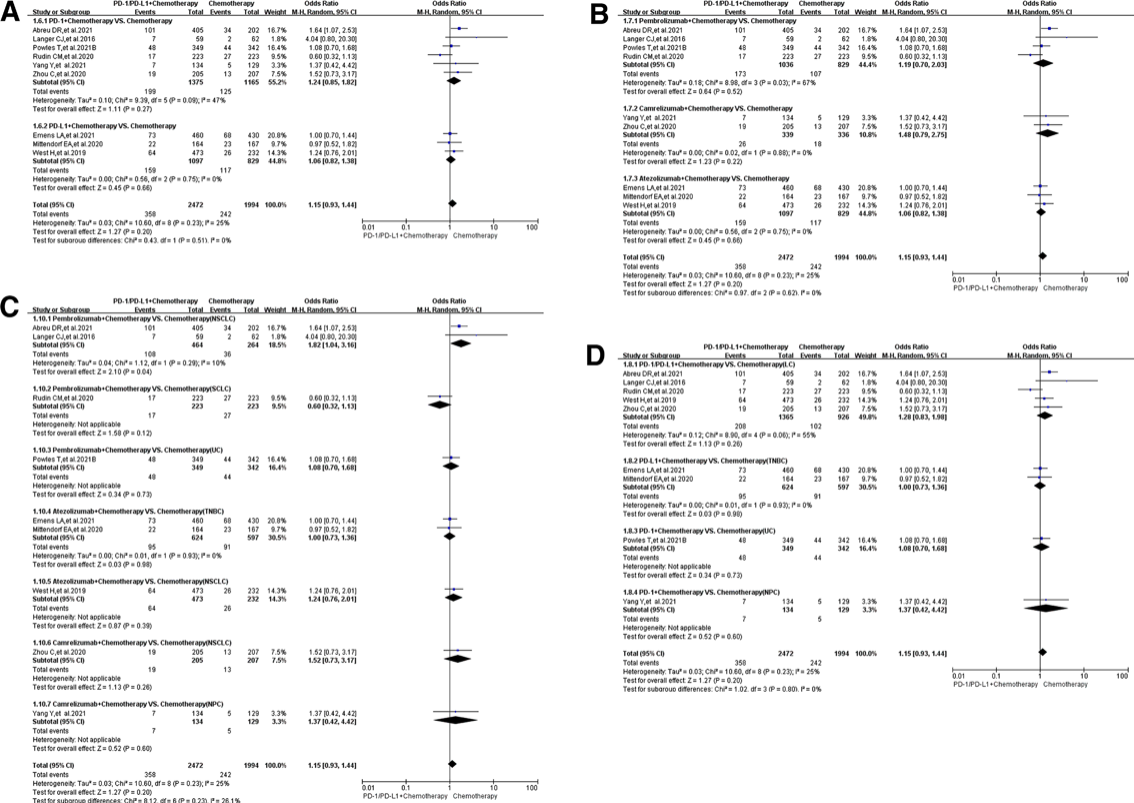
Forest plots of all-grade peripheral edema for group B (PD-1/PD-L1 + chemotherapy vs chemotherapy). (A) The risk of peripheral edema for all-grade evaluated by random effect model: subgroup analysis was carried out based on PD-1/PD-L1 inhibitors. (B) The risk of peripheral edema for all-grade evaluated by random effect model: subgroup analysis was carried out based on the specific name of PD-1/PD-L1 inhibitors. (C) The risk of peripheral edema for all-grade evaluated by random effect model: subgroup analysis was carried out based on specific immunosuppressive drugs and tumor types. (D) The risk of peripheral edema for all-grade evaluated by random effect model: subgroup analysis was carried out based on tumor type in the control group. PD-1 = programmed cell death-1, PD-L1 = programmed cell death ligand 1.

When PD-1/PD-L1 plus chemotherapy were compared with chemotherapy (Group B), no statistically significant difference regarding peripheral edema for all-grade could be found (OR = 1.15, 95% CI:[0.93, 1.44], *I*^2^ = 25%, *Z* = 1.27 (*P* = .20); Fig. 4A–D),^[17,20,21,23–26,30,31]^ even for every subgroup analysis results. A comprehensive review of the results of various subgroup analyses indicated that moderate heterogeneity might mainly be caused by the 2 clinical trials (KEYNOTE-361 and KEYNOTE-604) (Fig. 4B and C).^[17,31]^ No obvious publication bias could be found in the corresponding funnel plots (Supplemental Digital Content 2, http://links.lww.com/MD/H61).

### 3.5. The incidence risk of peripheral edema for grade 3–5

**Figure 5.**
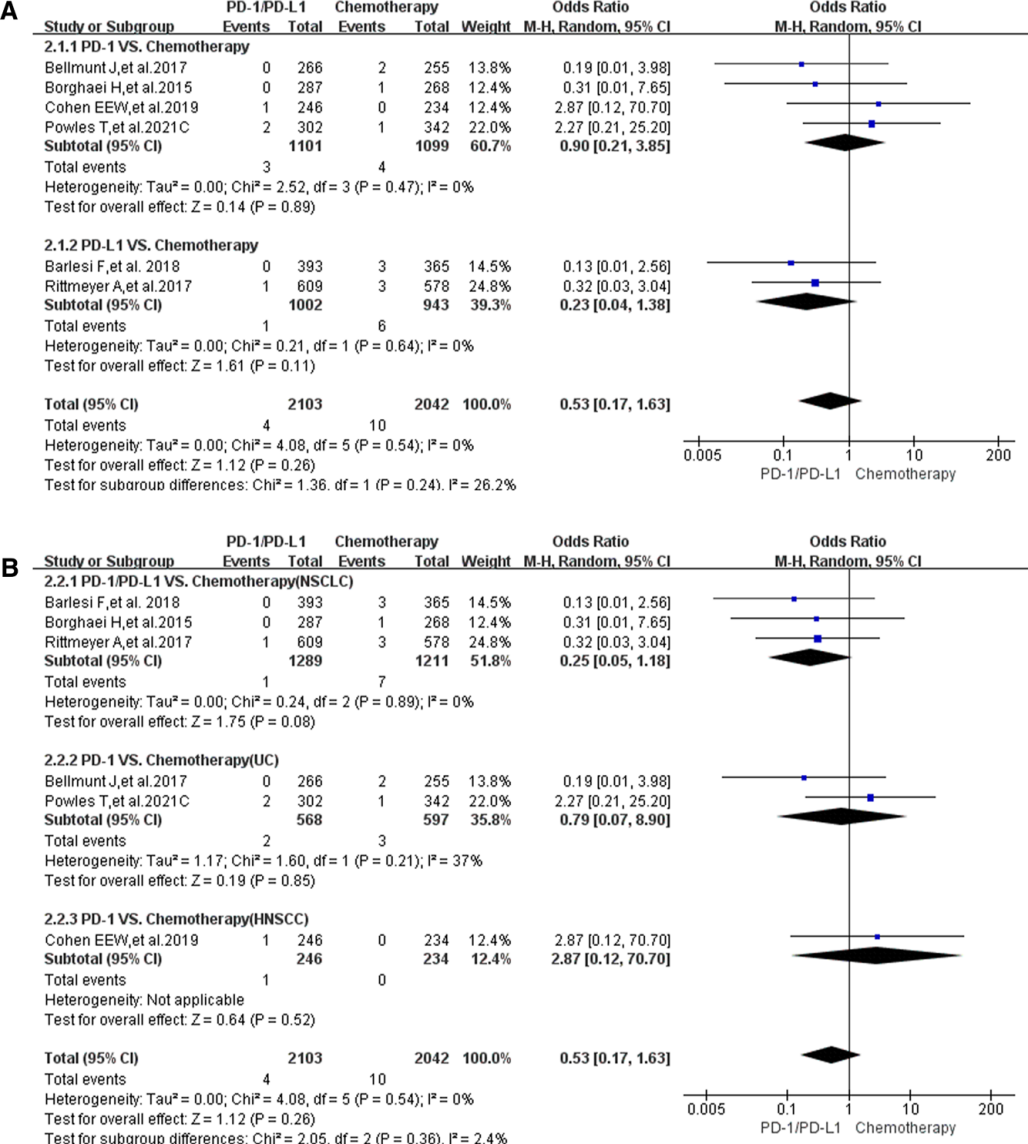
Forest plots of grade 3–5 peripheral edema for grade A. (A) The risk of peripheral edema for grade 3–5 evaluated by random effect model (PD-1/PD-L1 vs chemotherapy): subgroup analysis was carried out based on PD-1/PD-L1 inhibitors. (B) The risk of peripheral edema for grade 3–5 evaluated by random effect model (PD-1/PD-L1 vs chemotherapy): subgroup analysis was carried out based on tumor types. PD-1 = programmed cell death-1, PD-L1 = programmed cell death ligand 1.

The risk of developing peripheral edema for grade 3–5 was reported in 17 clinical trials,^[11,13,15,17–23,26,28,29,35,37–42]^ 10 of which were adopted for the final analysis^[11,13,15,17–21,23,26]^: Group A (PD-1/PD-L1 vs chemotherapy),^[11,13,15,17–19]^ and group B (PD-1/PD-L1 + chemotherapy vs chemotherapy).^[17,20,21,23,26]^ Compared with chemotherapy (group A), no statistically significant difference was found (OR = 0.53, 95% CI:[0.17, 1.63], *I*^2^ = 0, Z = 1.12 [*P* = .26]; Fig. 5A).^[11,13,15,17–19]^ Similar results could also be found in all the subgroup analyses (Fig. 5A and 5B). No heterogeneity (*I*^2^ = 0, Fig. 5) was found. The corresponding funnel plots were shown in the corresponding supplemental digital content (Supplemental Digital Content 3, http://links.lww.com/MD/H61).

**Figure 6.**
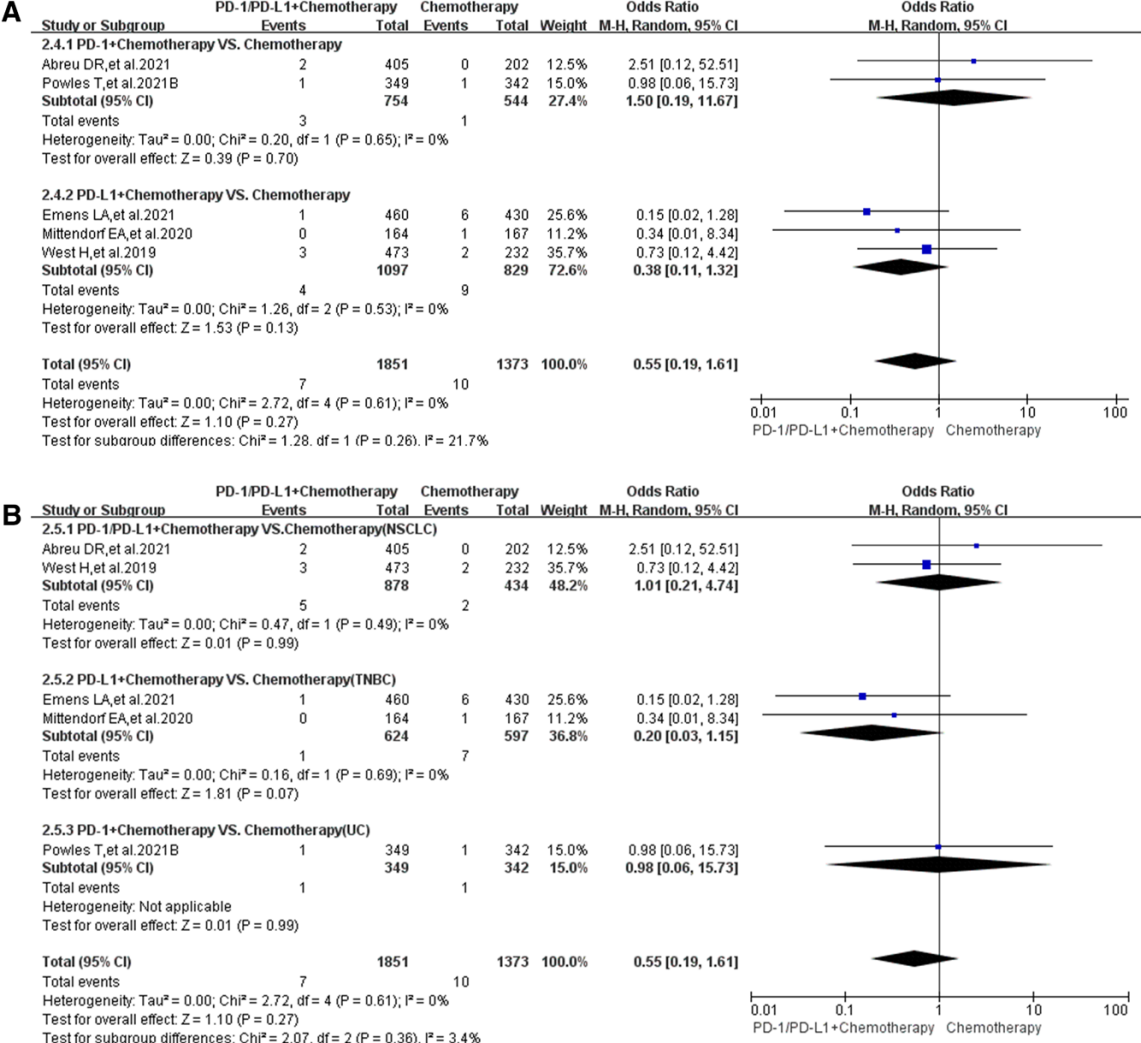
Forest plots of grade 3–5 peripheral edema for grade B. (A) The risk of peripheral edema for grade 3–5 evaluated by random effect model: subgroup analysis was carried out based on PD-1/PD-L1 inhibitors. (B) The risk of peripheral edema for grade 3–5 evaluated by random effect model: subgroup analysis was put into practice based on tumor types. PD-1 = programmed cell death-1, PD-L1 = programmed cell death ligand 1.

Similar results could also be found in Group B (PD-1/PD-L1 + chemotherapy vs chemotherapy). When PD-1/PD-L1 plus chemotherapy were compared with chemotherapy, no statistically significant results were noted (OR = 0.55, 95% CI: [0.19, 1.61], *I*^2^ = 0, *Z* = 1.10 [*P* = .27]; Fig. 6A),^[17,20,21,23,26]^ even in every subgroup analysis (Fig. 6B). No heterogeneity (*I*^2^ = 0) was found. No obvious publication bias was found in the corresponding funnel plots (Supplemental Digital Content 4, http://links.lww.com/MD/H61).

## 4. Discussion

Cancer immunotherapies, including ICIs and adoptive cell therapy, have transformed the treatment landscape for many types of tumors.^[1,8,50]^ With the rapid development of novel ICIs and combination treatment regimens, more and more clinical trials investigating immunotherapeutic drugs, especially for PD-1/PD-L1 inhibitors, have been conducted.^[11–42]^ An increasing number of adverse events, including peripheral edema, have also been reported.^[11–42]^ However, after searching online, we found that investigations on the risk of developing peripheral edema related to PD-1/PD-L1 inhibitors have rarely been conducted, and most of the peripheral edema events associated with PD-1/PD-L1 inhibitors were reported in the form of case reports.^[51–53]^ Therefore, those case reports on peripheral edema just had limited guidance significance for clinical works. For cancer patients, there are plenty of factors that can lead to peripheral edema, such as liver and kidney disease, chemotherapy, malnutrition, targeted therapy drugs and immunotherapy drugs, which further increases the difficulty of defining the cause of peripheral edema and giving clinical symptomatic treatment. In order to explore the relationship between PD-1/PD-L1 inhibitors and peripheral edema, this meta-analysis was designed and put into practice.

According to the guidelines of PRISMA, 22 clinical trials reporting peripheral edema events associated with PD-1/PD-L1 inhibitors, involving 15,233 cancer patients, were enrolled for the final analysis.^[11,13–21,23–26,30–42]^ Through the data on the basic characteristics of those clinical trials (Table 1), it could be found that the incidence rate of peripheral edema was not high, but it was an universal phenomenon. This also indicated that our analysis result was a concluding conclusion to a general problem, which might be much more representative than those case reports. In addition, the quality assessments of all enrolled clinical trials minimized the effect of bias caused by various reasons on the results (Fig. 2), thereby further increasing the reliability of the conclusion.

The analysis results showed that the risk of developing peripheral edema for all-grade caused by PD-1/PD-L1 inhibitors was significantly weaker than that of chemotherapy (Fig. 3); when they were combined with chemotherapy, there was a trend of increasing the risk of peripheral edema, but the analysis results were not statistically significant (Fig. 4). No statistically significant analysis results of peripheral edema for grade 3–5 was found, whether PD-1/PD-L1 was used alone or in combination with chemotherapy (Figs. 5 and 6). Those all indicated that PD-1/PD-L1 inhibitors had better safety and weaker toxicities.^[11–42]^ In conclusion, the effect of PD-1/PD-L1 inhibitor on the risk of developing peripheral edema was weaker than that of chemotherapy, and the combination with chemotherapy slightly increased the incidence risk of peripheral edema.

In some clinical trials, the incidence rate of PD-1/PD-L1-related peripheral edema was obviously higher than the placebo or observation group (Table 1),^[40,42]^ while it was lower than Sunitinib.^[39]^ However, since there were too few relevant clinical trials to conduct a meta-analysis, the conclusions were still controversial and needed to be further verified.^[39,40,42]^

To clarify the source of the different degrees of heterogeneity (Figs. 3 and 4), we performed adequate stratified subgroup analyses (Figs. 3–6). Although the results of the subgroup analysis suggested that the heterogeneity might be mainly derived from some clinical trials (Figs. 3 and 4),^[11–13,17,31]^ we combined the analysis results of all-grade and grades 3–5 and found that the heterogeneity might be mainly due to the data itself (Figs. 3–6). Furthermore, no obvious publication bias was found through funnel plots (Supplemental Digital Content 1 to 4, http://links.lww.com/MD/H61). In a word, the effect of heterogeneity and potential bias on the results had been minimized as much as possible.

Through our comprehensive analysis, it was clear that PD-1/PD-L1 slightly increased the risk of developing peripheral edema. Therefore, when encountering drug-induced peripheral edema in our clinical works, chemotherapy drugs should be considered first, followed by PD-1/PD-L1 inhibitors. For cancer patients with peripheral edema, we should also be alert to the risk of peripheral edema exacerbated by PD-1/PD-L1 inhibitors.

## 5. Conclusion

The effect of PD-1/PD-L1 inhibitor on the risk of developing peripheral edema was weaker than that of chemotherapy, and the combination with chemotherapy slightly increased the incidence risk of peripheral edema without statistical significance.

## Author contributions

The corresponding author (Yuan Tian) had the right to deal with all the data and was responsible for the decision to submit the manuscript for publication. A.H., M.T., K.W., and Q.D. had the data of all included clinical trials. C.Z., H.L., J.Z., X.Y., L.G., and C.Z. were responsible for checking and evaluating the quality of the collected data. F.C. was responsible for language revisions and English grammar proofreading.

## Supplementary Material


