# Supplementary material for: Incidence risk of peripheral edema in cancer patients treated with PD-1/PD-L1 inhibitors: A PRISMA guideline systematic review and meta-analysis
Source: Medicine (Baltimore). 2022 Sep 9;101(36):e30151. doi: 10.1097/MD.0000000000030151 (PMC10980461; doi:10.1097/MD.0000000000030151)

Supplemental digital content 1: Funnel plots of peripheral edema for any grade (Group A: PD-1/PD-L1 vs. Chemotherapy).

A: The risk of peripheral edema for all-grade evaluated by fixed effect model: subgroup analysis was carried out based on tumor types.

B: The risk of peripheral edema for all-grade evaluated by fixed effect model: subgroup analysis was carried out based on chemotherapy regimen (Docetaxel or Chemotherapy).

C: The risk of peripheral edema for all-grade evaluated by fixed effect model: subgroup analysis was carried out based on immunosuppressants types (PD-1 or PD-L1).

D: The risk of peripheral edema for all-grade evaluated by fixed effect model: subgroup analysis was carried out based on specific immunosuppressive drugs.

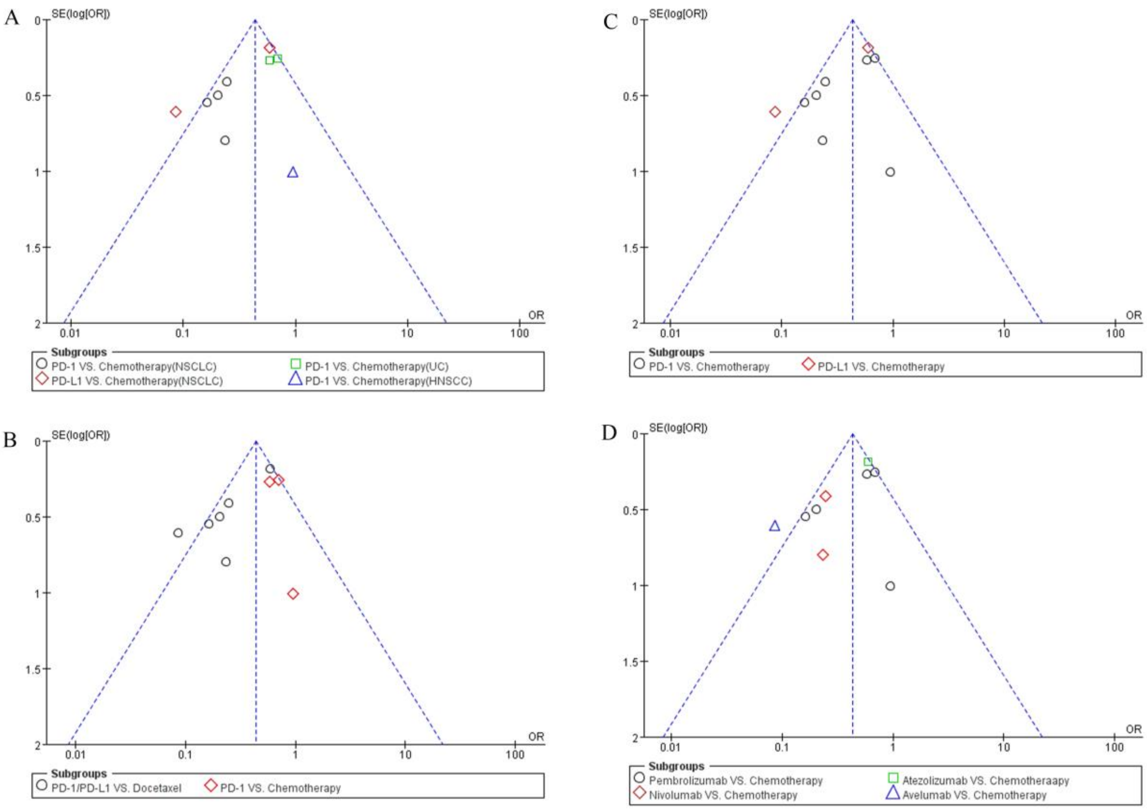

Supplemental digital content 2: Funnel plots of peripheral edema for any grade (Group B: PD-1/PD-L1+Chemotherapy vs. Chemotherapy)

A: The risk of peripheral edema for all-grade evaluated by fixed effect model: subgroup analysis was carried out based on PD-1/PD-L1 inhibitors.

B: The risk of peripheral edema for all-grade evaluated by fixed effect model: subgroup analysis was carried out based on the specific name of PD-1/PD-L1 inhibitors.

C: The risk of peripheral edema for all-grade evaluated by fixed effect model: subgroup analysis was carried out based on specific immunosuppressive drugs and tumor types.

D: The risk of peripheral edema for all-grade evaluated by fixed effect model: subgroup analysis was carried out based on tumor type in the control group.

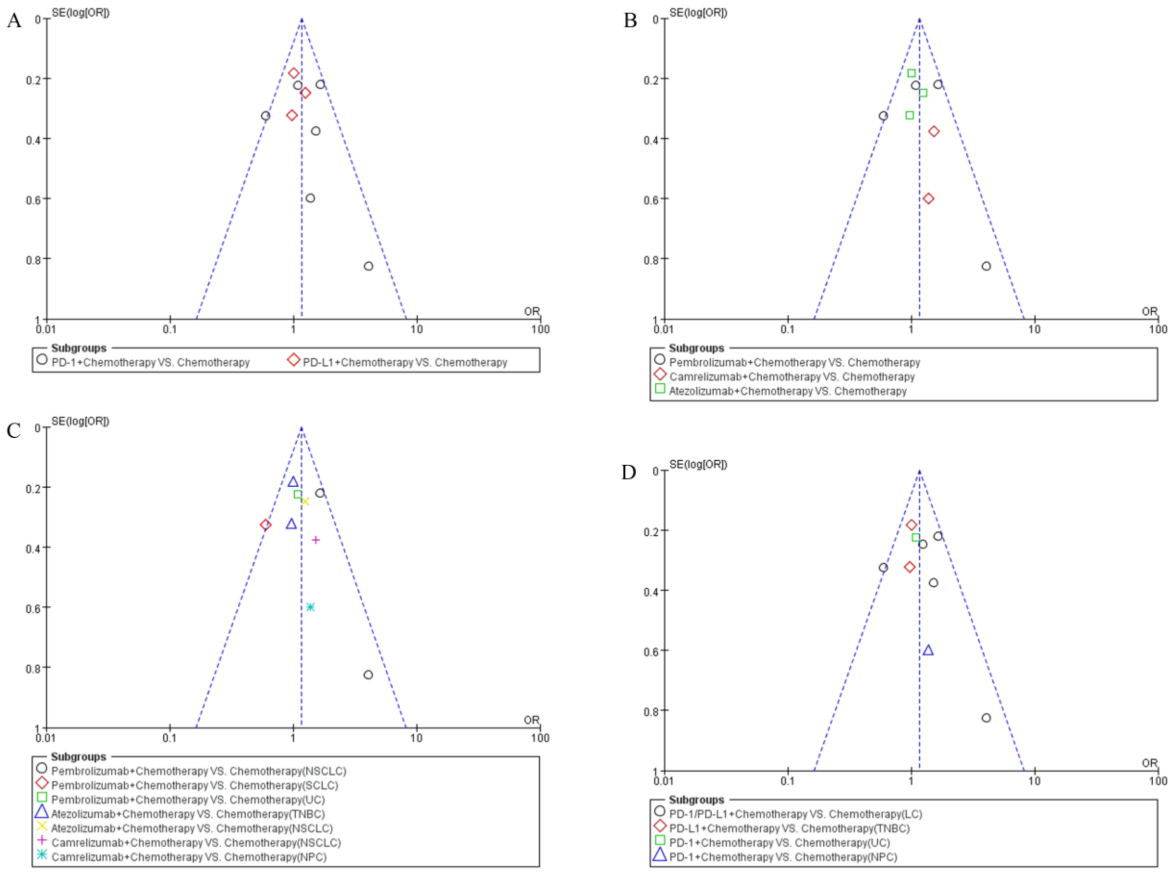

Supplemental digital content 3: Funnel plots of peripheral edema for grade 3-5 (Group A: PD-1/PD-L1 vs. Chemotherapy)

A: The risk of peripheral edema for grade 3-5 evaluated by fixed effect model: subgroup analysis was put into practice based on PD-1/PD-L1 inhibitors.

B: The risk of peripheral edema for grade 3-5 evaluated by fixed effect model: subgroup analysis was put into practice based on tumor types.

A

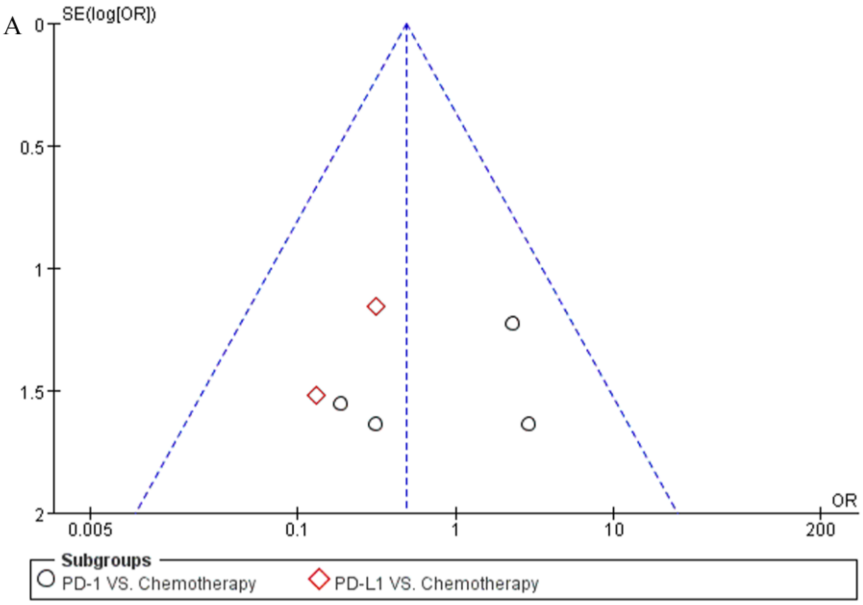

B

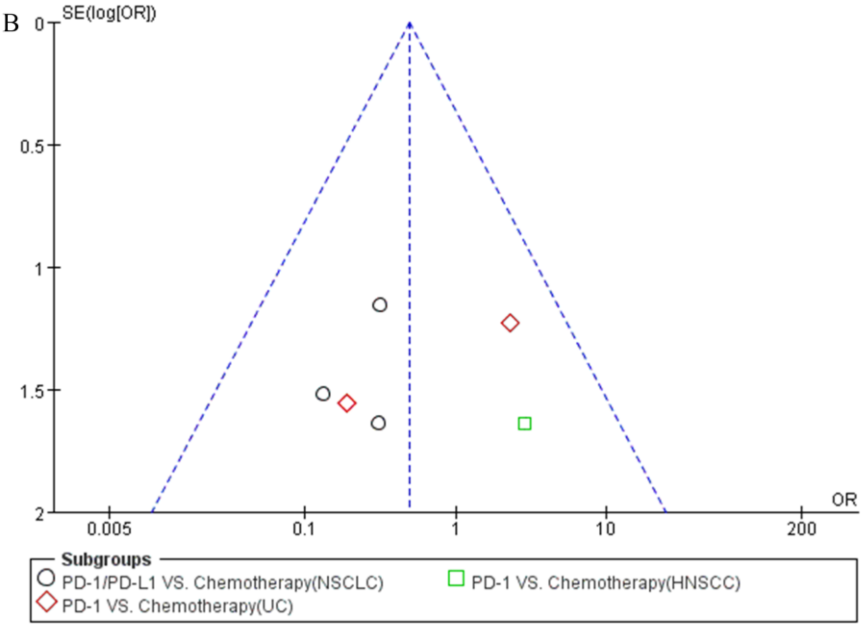

Supplemental digital content 4: Funnel plots of peripheral edema for grade 3-5 (Group B: PD-1 /PD-L1 + Chemotherapy vs. Chemotherapy)

A: The risk of peripheral edema for grade 3-5 evaluated by fixed effect model: subgroup analysis was put into practice based on PD-1/PD-L1 inhibitors.

B: The risk of peripheral edema for grade 3-5 evaluated by fixed effect model: subgroup analysis was put into practice based on tumor types.

A

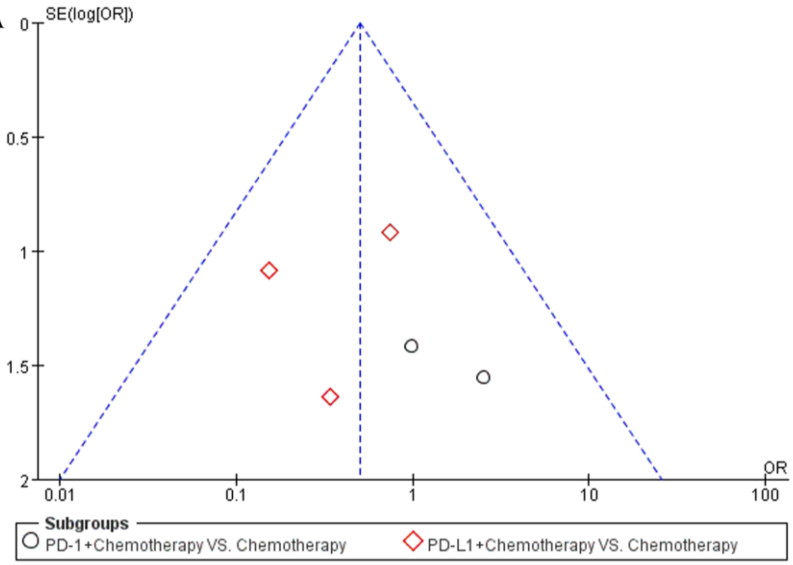

B

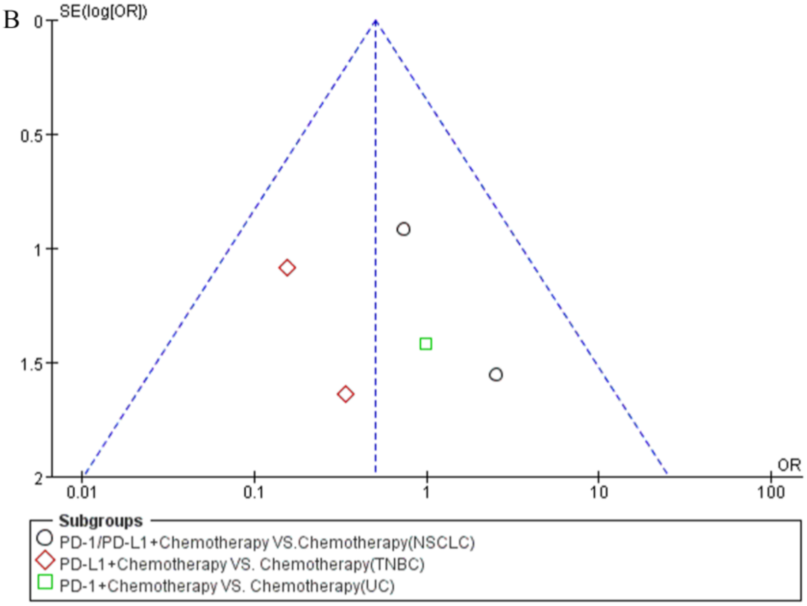

Supplement: Supplementary file 1 [file medi-101-e30151-s001.pdf]
